# Jujube Shell Based-Porous Carbon Composites Double-Doped by MnO_2_ and Ti_3_C_2_Tx: The Effect of Double Pseudocapacitive Doping on Electrochemical Properties

**DOI:** 10.3390/ma15217532

**Published:** 2022-10-27

**Authors:** Xue Sun, Qingwen Fan, Xiang Yin

**Affiliations:** College of Agricultural Engineering and Food Science, Shandong University of Technology, Zibo 255000, China

**Keywords:** biomass, porous carbon, MnO_2_, Ti_3_C_2_Tx, supercapacitor

## Abstract

In this study, manganese-containing porous carbon was synthesized from jujube shells by two-step carbonization and activation and was then covered with Ti_3_C_2_Tx to obtain double-doped biomass composites. In order to improve the interfacial properties (surface tension and wettability) between Ti_3_C_2_Tx and porous carbon, the effects of two media (deionized water and acetone solution) on the electrochemical properties of the composites were compared. The acetone solution changed the surface rheology of Ti_3_C_2_Tx and porous carbon, and the decreased surface tension and the increased wettability contributed to the ordered growth of 2D-Ti_3_C_2_Tx on the surface of the porous carbon. Raman analysis shows the relatively higher graphitization degree of JSPC&Ti_3_C_2_Tx (acetone). Compared with JSPC&Ti_3_C_2_Tx, JSPC&Ti_3_C_2_Tx (acetone) can maintain better rectangle-like properties even at a higher scanning rate. Under the effect of the acetone solution, the pseudocapacitive ratio of JSPC&Ti_3_C_2_Tx (acetone) increased from 10.1% to 30.7%. At the current density of 0.5 A/g, the specific capacitance of JSPC&Ti_3_C_2_Tx (acetone) achieved 96.83 F/g, and the specific capacitance of 58.17 F/g was maintained even at the high current density (10 A/g), which shows excellent magnification. Under the condition of the current density of 10 A/g, JSPC&Ti_3_C_2_Tx (acetone) can obtain a power density of 52,000 W/kg while maintaining an energy density of 8.74 Wh/kg. After 2000 cycles, the symmetrical button battery assembled with this material can still have a capacitance retention rate of more than 90%. This method realized the deep utilization of green and low-cost raw materials by using biomass as the precursor of composite materials and promoted the further development of carbon-based supercapacitor electrode materials.

## 1. Introduction

With the rapid development of modern society, the demand for energy is gradually increasing [1]. However, the limited reserves of fossil fuels are not enough to sustain the growing demand for energy, and there is an urgent need to develop clean and pollution-free renewable energy sources, such as wind energy, solar energy, tidal energy, biomass energy, nuclear energy, and others [2,3,4,5], and to commit to the development of corresponding energy storage and conversion technologies [6,7]. In the current energy storage technology, electrochemical energy storage is considered the most practical, advanced, and efficient technology, including fuel cells, supercapacitors, and metal ion batteries [8,9]. Supercapacitors usually have a low energy density, high power density, and long life, and their performance mainly depends on the physical and chemical characteristics of electrode materials.

In order to improve the electrochemical performance of electrode materials, it is necessary to synthesize and prepare new electrode materials according to the energy storage mechanism of supercapacitors. The energy storage process of supercapacitors mainly involves the double-layer capacitance process dominated by ion adsorption and desorption and the pseudo-capacitance process caused by redox reaction [10]. At present, carbon-based composites such as polymer, carbon nanotubes, and graphene show good electrochemical properties. Wang and Lu [11] have shown that a porous carbon composite was synthesized from alicyclic polyimides that exhibited a rich microporous/mesoporous structure and a large specific surface area. Moreover, only 0.1% of the capacity loss occurs after 10,000 cycles of charge and discharge. However, their high production cost hinders further practical application [12]. The production of biochar from biomass has attracted more and more attention due to its reusability, recyclability, and economy [13]. More importantly, biomass-derived carbon materials possess natural porosity, high specific surface area, and doped atoms, which contribute to electrolyte diffusion, shorter ion transport distances, and more active sites [14]. Consequently, biomass-based carbon materials and their composites are considered to provide high capacity, enhanced magnification, and enhanced cycle stability in electrochemical energy storage devices [7]. Ren and Yuan [15] have used aspen powder and carbon nanotubes to synthesize composites that show excellent electrochemical properties with a specific capacitance of 432.31 F/g at 0.5 A/g. In addition, Purkait and Singh [16] have obtained an energy density of 58.13 Wh/kg and a power density of 37.5 W/kg. The porous carbon prepared from shell biomass (peanut shell, coconut shell, etc.) has suggested excellent energy density and power density because the high specific surface area and large pore volume effectively enhance electrolyte ion penetration. The energy storage process of the carbon-based supercapacitors mainly came from the surface process of the electrode material, and the energy storage process was completed by the ion adsorption and desorption of the opposite charge. The process was a physical process that leads to the fast charging and discharging of carbon-based supercapacitors, which do not utilize continuous discharge applications. The energy storage mechanism of the pseudocapacitor is mainly related to the redox process of ions, and the sustainability of chemical reactions helps to ameliorate the disadvantage of the low energy density of supercapacitors [7,10]. Materials that can induce pseudocapacitive properties include metal oxides, conductive polymers, MXenes, organometallic framework MOFs, etc. [17,18]. In order to provide a high operating voltage window and high energy density, induced pseudocapacitive materials must possess multiple oxidation valences. MnO_2_ and Ti_3_C_2_Tx are excellent induced pseudocapacitive materials. MnO_2_ has a very high theoretical capacitance ranging from 1100 to 1380 F/g. The Faraday reaction of manganese oxide and the transition between different oxidation states in the redox ensure a high theoretical specific capacitance. However, due to various factors in practical application, the actual specific capacitance of MnO_2_ cannot reach the theoretical value. MnO_2_ exhibits strong pseudocapacitive behavior in electrolyte ion surface adsorption and fast reversible redox. A recently developed nanostructured MnO_2_ electrode has a specific capacitance of 500 F/g [19]. Nanosized MnO_2_ particles were combined with activated carbon, carbon nanotubes, or graphene to prepare composite materials, which obtained unexpected electrochemical properties [20]. Graphene-metal oxide composite presents a 3D graphene and MnO_2_ nanowires electrochemical synergistic effect to improve the energy storage capacity [21]. In recent years, novel materials including MXenes and metal-organic frameworks have attracted increasing attention in the field of pseudo-capacitor materials and their composites for high-performance capacitors. Wang and Zhang [22] have used a solvothermal method to synthesize three-dimensional nano-flowered Ni-MOF, which shows a high specific capacitance and excellent rate performance in supercapacitors because nanoscale flower-like structures provide fast ion transport channels and low resistance. MXenes have a great application prospect in the field of pseudocapacitor materials in energy storage since their first development [18]. Due to the high conductivity, well mechanical properties, and hydrophilicity of MXenes, it is considered a pseudocapacitor material that can obtain a high specific capacitance [23,24]. Ti_3_C_2_Tx-based composites could achieve a mass-specific capacitance of up to 245 F/g, exhibiting excellent rate performance, and cyclic stability [23]. MXenes/CNT composites show better rate properties, which contribute to improved energy density properties at high power densities compared with pure Ti_3_C_2_Tx [24].

Therefore, we used MnO_2_ as a primary pseudocapacitive inducer and biomass to prepare porous carbon composites containing manganese metal. Then the Ti_3_C_2_Tx material was grown on the surface of the composite in different media to make the composite with double pseudocapacitive doping so as to improve the pseudocapacitive characteristics of the electrode material. The prepared electrode material shows excellent pseudocapacitive characteristics, and the specific capacitance stability of 2000 cycles is maintained above 90%.

## 2. Materials and Methods

### 2.1. Materials

A total of 60% PTFE (D21C, DAIKIN, Osaka, Japan) emulsion was diluted to 10% for standby. Conductive carbon black (BP2000, CARBOT, Boston, MA, USA) was dried for 12 h. The foamed nickel (Changde Liyuan New Materials Co., Ltd., Changde, China) was ultrasonically cut into 18 × 18 mm sheets in anhydrous ethanol medium for 30 min, with the weight recorded as m_1_. Ti_3_C_2_Tx powder was purchased from the *11 Technology Co., Ltd*. (Changchun, China), which is a two-dimensional multi-layered structure. All chemical reagents used in this experiment were analytical reagent grade.

### 2.2. Experimental Methods

The raw materials of the jujube shell were crushed, soaked in 1.0 M sulfuric acid, stirred for 24 h, and then dried. Then they were heated to 400 °C at 5 °C/min in a tube furnace with a nitrogen atmosphere for 2 h. The solid product was thoroughly ground through a standard sieve of 200 mesh. Subsequently, MnO_2_ as Metal Oxide Dopant was mixed in deionized water according to the mass ratio (charred jujube shell: MnO_2_ = 1:2) to make the suspension. It was heated to 1000 °C at 5 °C/min for 60 min in a tube furnace. The carbon material was completely immersed in 2 M HCl solution and stirred for 10 h (to remove inorganic impurities, metal salts, and ash to increase porosity) and washed to neutral with deionized water. The sediment was dried at 105 °C and ground through the sieve of 300 mesh.

The activated porous carbon material and Ti_3_C_2_Tx powder were mixed in 60 mL of deionized water at the ratios of 9:1, 8:2, and 6:4 to form a suspension. The film was sealed and stirred at room temperature for 12 h. The supernatant was removed by centrifugation and the solid residue was dried in a vacuum for 24 h to obtain the composite carbon materials, which were named JSPC&Ti_3_C_2_Tx-1, JSPC&Ti_3_C_2_Tx-2, and JSPC&Ti_3_C_2_Tx-3, respectively.

In order to improve the infiltration between porous carbon and Ti_3_C_2_Tx, the medium water was replaced by an acetone solution (50%). The porous carbon material and Ti_3_C_2_Tx powder were mixed in 60 mL acetone solution according to the ratio of 9:1 to form a suspension. The film was sealed, and after magnetic stirring for 12 h, the supernatant was removed, and the solid residue was dried in a vacuum for 24 h. The composite carbon material is named JSPC&Ti_3_C_2_Tx (acetone).

### 2.3. Structural Characterizations

The surface morphology and microstructure of the composite were observed by scanning electron microscope (SEM, Quanta 250, FEI, Hillsboro, OR, USA) and transmission electron microscope (TEM, Tecnai G^2^ F20, FEI, American). Confocal Raman spectroscopy (Raman, LabRAM HR Evolution, HORIBA JobinYvon, Palaiseau, France) was used to characterize the defect extent of carbon materials in the wavelength range of 100–3000 cm^−1^ with He-Ne laser 633 as the excitation wavelength. In order to clarify the kinds of elements and their valency distribution on the surface of the composite materials, the alk α was used as the excitation source for the calibration by X-ray Photoelectron Spectrometer (XPS, Nexsa, Thermo Fisher, Waltham, MA, American). Fourier infrared spectroscopy analysis (FT-IR, ALPHA II, BRUKER, Ettlingen, Germany) was used to analyze the surface functional groups of porous carbon materials in the range of 400–4000 cm^−1^.

### 2.4. Electrochemical Measurements

#### 2.4.1. Electrode Sheet Preparation

Electrode material: conductive carbon black: PTFE = 8:1:1 were mixed and the right amounts of anhydrous ethanol were added in the full grinding, the rubber clay-like mixture. The mixture was repeatedly folded and rolled by a glass rod to improve the dispersion uniformity and the stacking density of the mixture. The carbon film was spread on the weighing paper and dried in an 80 °C vacuum oven for 8 h, and a 15 × 15 mm square electrode slice and d = 10 mm round electrode slice were cut out. The cut electrode plates were laid flat on the weighing nickel foam for 10 s at 20 MPa, and the weight was recorded as m_2_. The mass of the active substance of the electrode material was recorded as m = (m_2_ − m_1_) × 0.8.

#### 2.4.2. Three Electrode System

The prepared electrode was soaked in 1 M KOH electrolyte for 8 h. CV (5, 10, 20, 50, 80, and 100 mV/s) and GCD (0.5, 1, 2, 5, 8, and 10 A/g) were measured at an electrochemical workstation (PGSTAT 302N, Autolab, Switzerland) using a 1 M KOH electrolyte and a mercuric oxide reference electrode. EIS was tested using an electrochemical workstation (Zahner, Zennium Pro, Kronach, Germany). The pseudocapacitance was obtained by fitting the cyclic voltammetry curve [25].

The mass-specific capacitance (*C*, F/g) was calculated based on GCD data,

*C* = (*I Δt*)/(*m ΔV*) (1)

*I*: discharge current, *Δt*: discharge time, *m*: the mass of the active substance in a single electrode (g), *Δt*: the potential difference after IR drop is removed in the discharge process.

The energy density (*E*, W h/kg) and the power density (*P*, W/kg) referred to the following formulas respectively:

*E* = (*C ΔV*^2^)/7.2 *P =* 1000 *E*/*t* (2)

#### 2.4.3. Two Electrode System

A symmetrical button battery was mounted on two circular electrode plates of the same mass of the active substance. After the button battery was left for 8 h, the electrochemical performance of the device was tested in the electrochemical workstation, these include CV (scan rate of 5, 10, 20, 50, 80, and 100 mV/s), GCD (current density of 0.5, 1, 2, 5, 8, and 10 A/g), EIS, and 1 A/g current density to measure the cyclic stability of button cells (2000 cycles).

## 3. Results and Discussion

### 3.1. Characteristics of Composites

**Figure 1:**
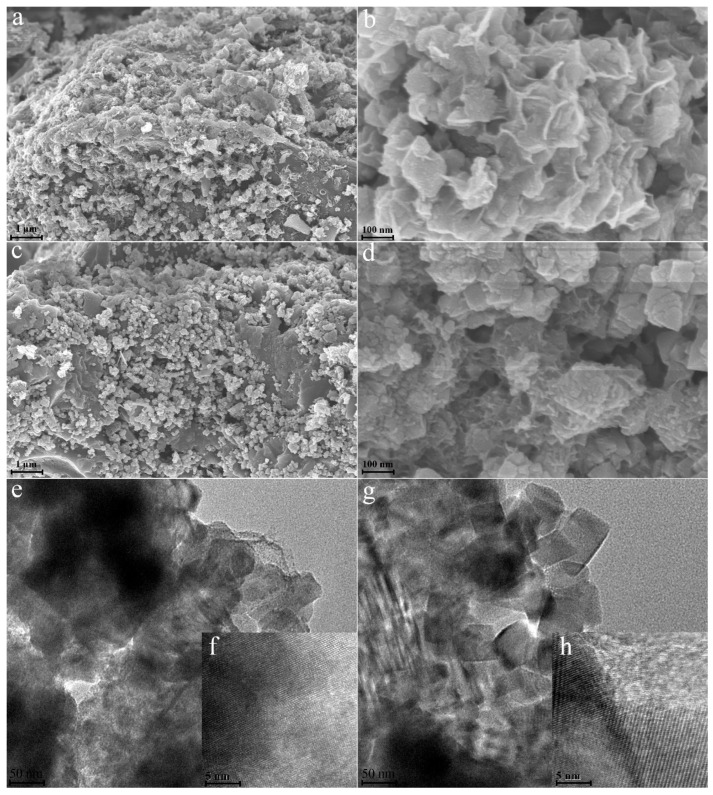
Morphology characteristics of porous carbon&Ti_3_C_2_Tx Composites. A, b, e, and f represent the deionized water as the medium ((**a**,**b**) refer to SEM images, (**e**,**f**) refer to TEM images.). (**c**,**d**,**g**,**h**) represent the acetone as the medium (**c**,**d**) refer to SEM images, (**g**,**h**) refers to TEM images).

Figure 1 shows the morphology characteristics of composite materials in different solution media. It is observed that Ti_3_C_2_Tx was successfully coated on the surface of the carbon material whether the medium was deionized water or acetone (Figure 1a,c). The difference lies in the disordered growth of Ti_3_C_2_Tx on the surface of porous carbon in the presence of deionized water, no matter the particle size or the growth direction. It is well known that MXenes is a two-dimensional layered structure, and Ti_3_C_2_Tx was found to have a disordered arrangement in both transverse and longitudinal directions on the porous carbon surface from Figure 1b. The acetone solution as the medium was helpful to the orderly growth of Ti_3_C_2_Tx material on the carbon surface showing a more uniform particle distribution and orderly direction (Figure 1c,d). As a polar organic substance, acetone would reduce the surface tension of water when it was dissolved in water. After Huang, Chu [26] added acetone to the water, the reduction of contact angle proved the reduction of surface tension. The reduced surface tension improved the surface wettability of carbon and made the adhesion between the carbon surface and the MXenes layer stronger. Figure 1d shows the morphological characteristics of a sheet-like stack, which is consistent with the two-dimensional multi-layer structure of Mxenes. The surface rheology of Mxenes was changed by an acetone solution [26]. The MXenes aqueous solution usually has problems of high surface tension and poor wettability. After adding acetone, the surface tension of the water was reduced, and the wettability of the material was improved. This allowed the layered MXenes material to better match the porous carbon surface along the transverse direction and to have good adhesion. According to the morphology and structure of TEM, it can also be observed that the layered structure of Ti_3_C_2_Tx in acetone solution was obviously superior to that in deionized water (Figure 1e,g). In addition, the TEM images of the composites showed obvious lattice fringes. The lattice structure helped to improve the conductivity and charge transfer rate of the carbon materials (Figure 1f,h).

**Figure 2:**
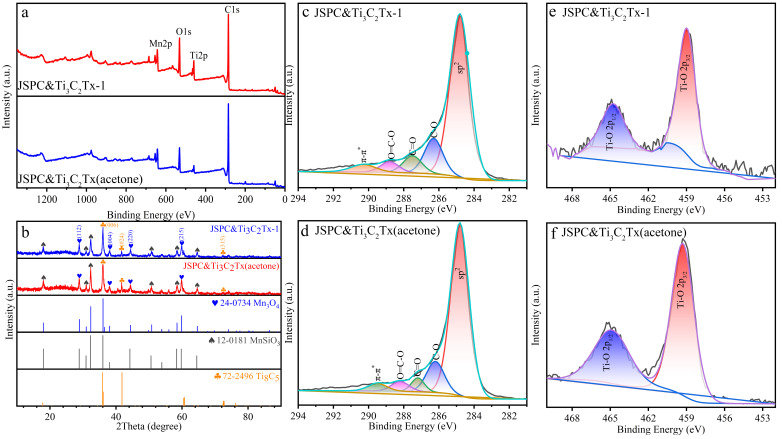
XPS survey spectra (**a**), XRD patterns (**b**) and high-resolution spectrum ((**c**,**e**): C1s and Ti 2p of JSPC&Ti_3_C_2_Tx-1; (**d**,**f**): C1s and Ti 2p of JSPC&Ti_3_C_2_Tx (acetone)).

**Figure 3:**
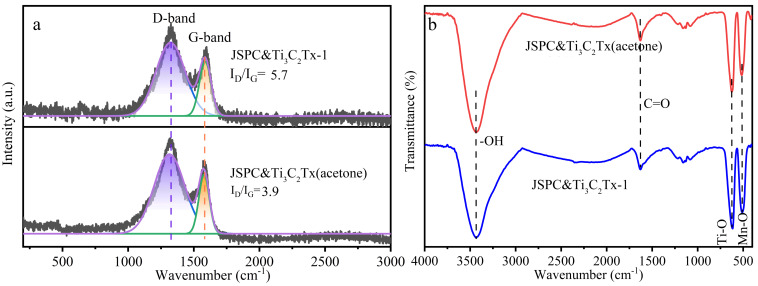
Raman (**a**) and FTIR (**b**) spectrum of porous carbon composites.

In order to clearly understand the elemental composition and chemical bonding state of porous carbon composite MXenes, the composite was characterized by XPS. According to the survey spectrum of XPS (Figure 2), the composite was mainly composed of C, O, Ti, and Mn elements. The C1s element was the dominant element category, followed by O1s (Table 1). The content of Mn2p in the two materials was the same. More importantly, the proportion of Ti2p in JSPC&Ti_3_C_2_Tx (acetone) was much higher than that in JSPC&Ti_3_C_2_Tx-1, indicating that the acetone solution changed the interface effect between MXenes and porous carbon, the lower surface tension increased the adhesion between Ti_3_C_2_Tx and porous carbon [26]. The high-resolution spectra of C1s in the composites were located at the peak positions of 284.8, 286.3, 287.5, 288.8, and 290.3 eV, corresponding to the sp^2^ hybrid carbon (C=C/C-C), C-O, C=O, O=C-O, π-π *, respectively [27]. The similar distribution of JSPC&Ti_3_C_2_Tx-1 and JSPC&Ti_3_C_2_Tx-1 in C1s showed that acetone solution does not change the molecular structure of the material, but only affects the physical properties of its surface. The strongest peak of the sp^2^ hybrid carbon at 284.8 eV indicated that graphite carbon was the main component of porous carbon, whether the medium was water or acetone. Two strong characteristic peaks (Ti-O 2p_1/2_ and Ti-O 2p_3/2_) were detected in the high-resolution spectra of Ti 2p, and the binding energy peaks were 459.3 eV and 465.1 eV, which corresponded to the Ti-O Bond. The increase of the Ti-O peak in the high-resolution spectra may be due to the oxidation of the Ti_3_C_2_ multilayer material surface [28]. The binding energy of Ti 2p high-resolution spectra in the JSPC&Ti_3_C_2_Tx (acetone) material had a slight negative shift compared with that of JSPC&Ti_3_C_2_Tx-1 from Figure 2e,f. This might be because the acetone medium reduced the binding energy of Ti 2p, making the atomic proportion of Ti 2p in JSPC&Ti_3_C_2_Tx (acetone) much higher than JSPC&Ti_3_C_2_Tx-1 (Table 1). Figure 2b describes the XRD patterns of JSPC&Ti_3_C_2_Tx-1 and JSPC&Ti_3_C_2_Tx (acetone), showing obvious diffraction peaks with the crystalline state [29]. Combined with JCPDS card information, three phases were found in the composites, namely Mn_3_O_4_ (24-0734), MnSiO_3_ (12-0181), and Ti_8_C_5_ (72-2496). The diffraction peak positions of XRD patterns of JSPC&Ti_3_C_2_Tx-1 and JSPC&Ti_3_C_2_Tx (acetone) showed a high consistency, which suggested that the acetone solution had no effect on the crystal state. Figure 3a is the Raman spectrum of the composite material, which showed the graphitization degree and carbon defect degree of the porous carbon material. JSPC&Ti_3_C_2_Tx (acetone) and JSPC&Ti_3_C_2_Tx-1 presented two characteristic peaks at 1350 cm^−1^ (D-band) and 1580 cm^−1^ (G-band). The D peak represented the disordered carbon atoms at the carbon defect site or sp^2^ hybrid, and the G peak represented the in-plane vibration of sp^2^ heteroatoms in the carbon material [30]. *I*_D_/*I*_G_ is commonly used to reflect the degree of graphitization or the degree of carbon defects in porous carbon materials [31], that is, a smaller *I*_D_/*I*_G_ value indicates a higher degree of graphitization, and a larger *I*_D_/*I*_G_ value indicates a lower degree of carbon defects. The *I*_D_/*I*_G_ value of JSPC&Ti_3_C_2_Tx (acetone) was significantly lower than that of JSPC&Ti_3_C_2_Tx-1, which indicated a relatively high degree of graphitization, consistent with the high-resolution C1s profile in the XPS results (Figure 1b). Figure 3b reflects the FTIR spectrum of composite materials, and JSPC&Ti_3_C_2_Tx (acetone) and JSPC&Ti_3_C_2_Tx-1 showed common characteristic peaks. The wide peak at 3440 cm^−1^ corresponded to the stretching vibration of OH. The weak vibration at 1628 cm^−1^ is attributed to the telescopic vibration of C=O. The spikes at 618 cm^−1^ and 510 cm^−1^ were caused by vibrations of Ti-O and Mn-O [32,33].

### 3.2. Electrochemical Properties of Composites

**Figure 4:**
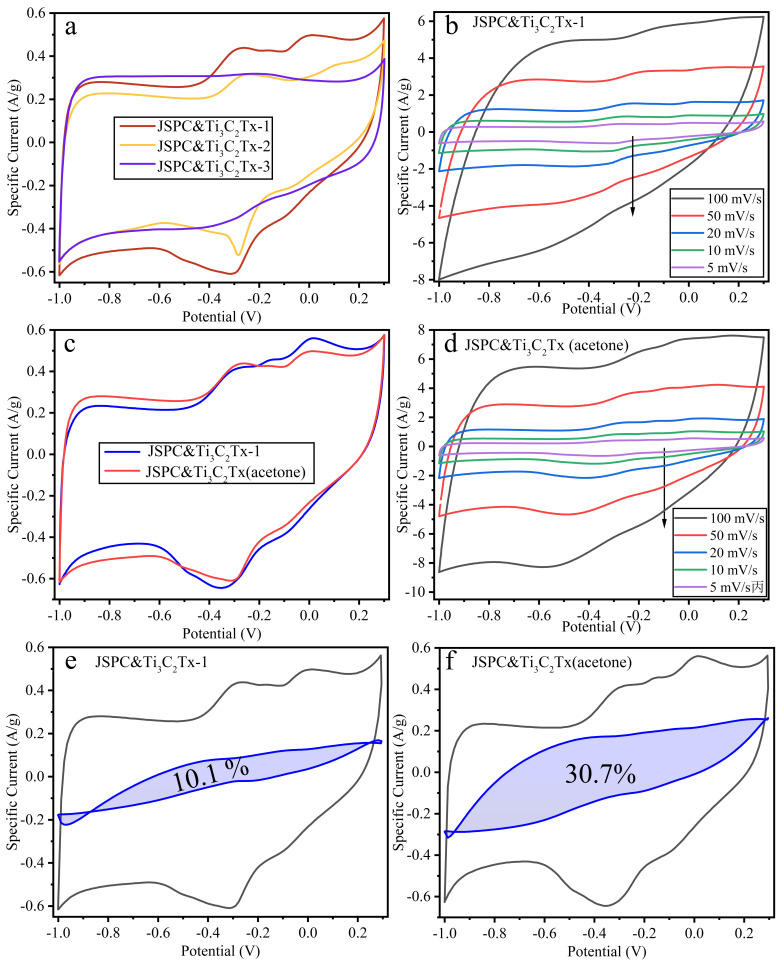
Comparison of CV curves and the proportion of pseudocapacitors in the composites. (**a**) is the CV curves of JSPC&Ti_3_C_2_Tx at 5 mV/s. (**b**) refers to the CV curves of JSPC&Ti_3_C_2_Tx at several scanning speeds. (**c**) is the CV curves of JSPC&Ti_3_C_2_Tx-1 and JSPC&Ti_3_C_2_Tx(acetone) at 5 mV/s. (**d**) is the CV curves of JSPC&Ti_3_C_2_Tx(acetone) at several scanning speeds. (**e**,**f**) refer to the CV and pseudocapacitance curves of JSPC&Ti_3_C_2_Tx-1 and JSPC&Ti_3_C_2_Tx(acetone) at 5 mV/s.

**Figure 5:**
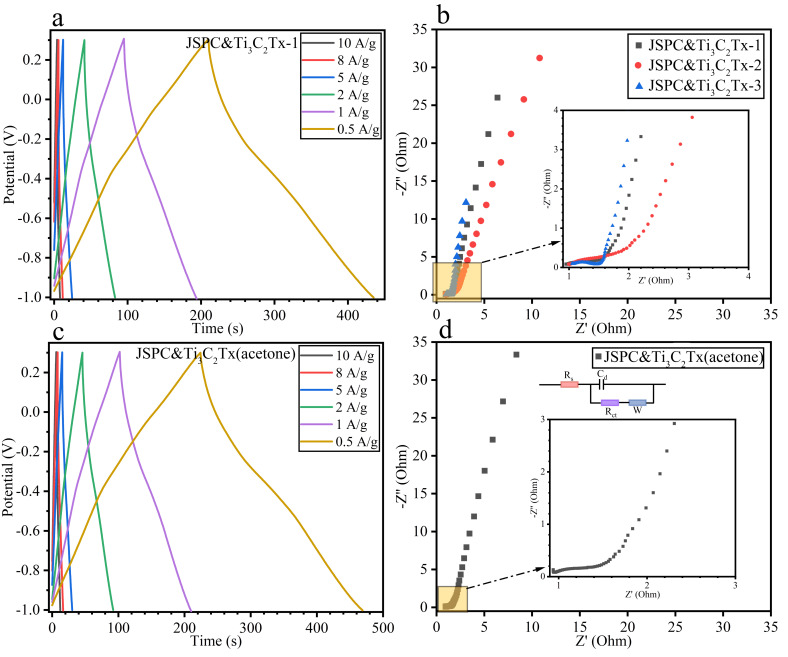
GCD and EIS curves of the composites. (**a**,**c**) refer the GCD curves of JSPC&Ti_3_C_2_Tx-1 and JSPC&Ti_3_C_2_Tx(acetone). (**b**,**d**) refer to EIS and equivalent current diagram.

Figure 4a describes the CV curve profile of the composite prepared by mixing manganese-containing porous carbon with Ti_3_C_2_ in deionized water at different ratios at a scanning speed of 5 mV/s. It was found that the low Ti_3_C_2_ content could improve the specific capacitance of the composites, based on the CV curve integral area data. This might be because the appropriate Ti_3_C_2_ content helped to cover the carbon surface evenly, while the excessive Ti_3_C_2_ content led to the collapse of the multilayer structure, which is detrimental to ion storage [34]. Then the gradient sweep velocity of the JSPC&Ti_3_C_2_Tx-1 composite was measured (Figure 4b). It was found that the CV curve maintained a good rectangle-like shape at low sweep velocity, while the high sweep velocity distorted the rectangle-like shape of the CV curve. This reflected the poor double-layer capacitance of the composite material. In order to change the interface characteristics between porous carbon and Ti_3_C_2_Tx multilayer materials, the deionized water was replaced by an acetone solution. The mass-specific capacitance of the composites obtained under the two media conditions possessed similarity based on the CV curve data from Figure 4c. In the acetone solution, the position of the oxidation peak of the CV curve was obviously deviated, especially the position of the third oxidation peak. Moreover, the width of the reduction peak is much wider than that of JSPC&Ti_3_C_2_Tx-1. The multistage scanning velocity of the JSPC&Ti_3_C_2_Tx (acetone) composite was measured (Figure 4d). It can be found that the CV curve modified by acetone solution could keep a rectangle-like characteristic even at high sweeping speed, which showed that the stability of electric double-layer capacitor had been improved. The main reason was that the acetone solution reduced the surface tension between porous carbon and Ti_3_C_2_ multilayer materials and improves the wettability and the adhesion of the interface. Pseudocapacitive fitting was performed on JSPC&Ti_3_C_2_Tx-1 and JSPC&Ti_3_C_2_Tx (acetone) based on the CV curve of 5 mV/s ((Figure 4e,f). It can be seen that the ratio of pseudocapacitance of JSPC&Ti_3_C_2_Tx-1 is only 10.1%. Interestingly, the pseudocapacitive ratio of JSPC&Ti_3_C_2_Tx (acetone) increases to 30.7% after treatment with the acetone solution, which indicated that Ti_3_C_2_ is a good material for inducing pseudocapacitance. Figure 5 describes the GCD and EIS curves of JSPC&Ti_3_C_2_Tx-1 and JSPC&Ti_3_C_2_Tx (acetone). Figure 5a,c can be comparatively analyzed, and both composites have good symmetry and linear independence, which indicates their charge-discharge reversibility was well. The charging and discharging time of JSPC&Ti_3_C_2_Tx (acetone) was slightly longer than that of JSPC&Ti_3_C_2_Tx-1, which indicated that the mass-specific capacitance of the composite was slightly improved by acetone solution treatment. Under the current density of 0.5 A/g, the mass-specific capacitance of JSPC&Ti_3_C_2_Tx (acetone) reaches 96.83 F/g, and the mass-specific capacitance of 58.17 F/g is maintained even under the condition of high current density (10 A/g). According to Table 2, energy density and power density are two opposing parameters, with energy density negatively correlated and power density positively correlated with current density. At a current density of 10 A/g, the power density of JSPC&Ti_3_C_2_Tx (acetone) reaches 52,000 W/kg, which can be used in high power demand. However, at 10 A/g, JSPC&Ti_3_C_2_Tx (acetone) got an energy density of 8.74 Wh/kg, which was 94.22% higher than that of JSPC&Ti_3_C_2_Tx-1 under the same conditions. Although the specific capacitance of JSPC&Ti_3_C_2_Tx (acetone) and JSPC&Ti_3_C_2_Tx-1 is lower than that of other electrode materials, they exhibit higher energy density and power density under the same conditions (Table 2). The EIS impedance spectra of JSPC&Ti_3_C_2_Tx showed that the addition of Ti_3_C_2_Tx had a lower effect on the intrinsic resistance of the composites, and their internal resistance was less than 1 Ω (Figure 5b), indicating that JSPC&Ti_3_C_2_Tx-MnO_2_ had the best intrinsic conductivity. Near arc in high-frequency region of EIS. The EIS of all materials is approximately a straight line and the slope is more than 45° in the low frequency region, which indicates a good capacitive storage mechanism. The porous nature of the composites contributes to efficient electron and ion transport. The EIS spectra of JSPC&Ti_3_C_2_Tx (acetone) had similar characteristics (Figure 5d).

### 3.3. Electrochemical Properties of the Cell

**Figure 6:**
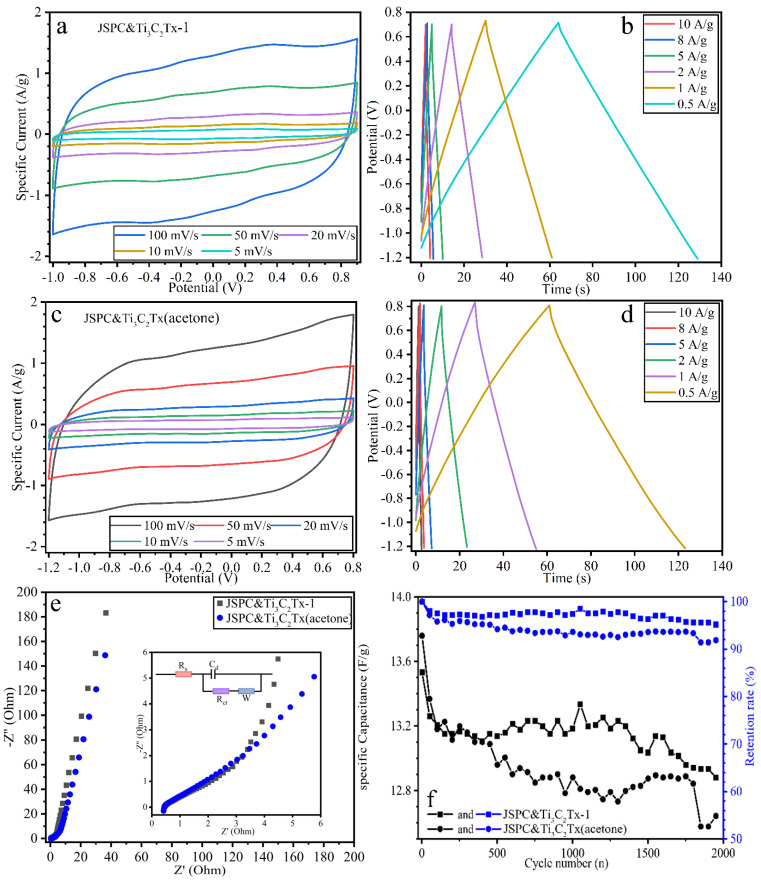
Electrochemical characteristics of the device. (**a**,**b**) refer to the CV and GCD from JSPC&Ti_3_C_2_Tx-1. (**c**,**d**) refer to the CV and GCD from JSPC&Ti_3_C_2_Tx (acetone). (**e**) is the EIS curve. (**f**) is the cyclic stability.

Figure 6a,c describe the CV curve of the symmetrical button cell composed of JSPC&Ti_3_C_2_Tx-1 in 1 M KOH electrolyte with different sweep velocities. The voltage range of the cell is −1~+0.8 V, and JSPC&Ti_3_C_2_Tx (acetone) material extends the voltage range to −1.2~0.8 V. The CV curve has a nearly perfect central symmetry, which indicates a high degree of redox. Figure 6b,d reflects the GCD curve characteristics of the device assembled by two kinds of composite materials in 1 M KOH electrolyte at different current densities. It can be seen that the charge and discharge process of the GCD was highly symmetrical and had linear independence. The GCD curve characteristics of the device assembled by two kinds of composite materials in 1 M KOH electrolyte at different current densities were reflected, and the charge and discharge processes of the GCD are highly symmetrical and have linear independence. Figure 6e reflects the EIS curve of the device. The two devices have a lower inherent resistance (about 0.5 Ω), indicating that the device composed of two materials has the best conductivity. The curve of EIS is nearly a straight line, and the slope is more than 1 in the low-frequency region, which indicates a well-capacitive energy storage mechanism [39]. Under the current density of 1A/g, the mass-specific capacitance of JSPC&Ti_3_C_2_Tx-1 and JSPC&Ti_3_C_2_Tx (acetone) reach 13.53 and 13.76 F/g, respectively. Over the course of 2000 cycles (Figure 6f), it can be found that the mass capacitance ratio of both materials decreases rapidly at the beginning of the long cycle, which may be due to the structural instability of the composites at the initial stage. The cycle stability of device JSPC&Ti_3_C_2_Tx-1 is better than that of device JSPC&Ti_3_C_2_Tx(acetone). After 2000 charge–discharge cycles, the capacitance retention rate of the JSPC&Ti_3_C_2_Tx-1 device is over 94%, while that of the JSPC&Ti_3_C_2_Tx(acetone) device is only over 90%.

## 4. Conclusions

A two-step method of carbonization and activation was used to obtain the composite material of the jujube shell. MnO_2_ can be used as both an activator and a catalyst to reduce the graphitization temperature during the synthesis process. The obtained JSPC&Ti_3_C_2_Tx (acetone) composites showed a high pseudocapacitive proportion (30.7%). JSPC&Ti_3_C_2_Tx (acetone) exhibits excellent electrochemical performance, with a mass-specific capacitance of 96.83 F/g at the current density of 0.5 A/g. Under the condition of high current density (10A/g), which keeps the mass ratio capacitance of 58.17 F/g, it showed excellent multiplicity. Under the current density of 10 A/g, the power density of 52,000 w/kg and the energy density of 8.74 Wh/kg are obtained from JSPC&Ti_3_C_2_Tx (acetone). After 2000 cycles of charging and discharging, the symmetrical button battery still has a capacitance retention rate of more than 90%.

## Figures and Tables

**Table 1:** The atomic ratio in the composites from XPS data.

Atomic (%)	C1s	O1s	Ti2p	Mn2p
JSPC&Ti_3_C_2_Tx-1	79.98	13.75	2.04	4.23
JSPC&Ti_3_C_2_Tx (acetone)	72.84	17.94	5.03	4.2

**Table 2:** The mass-specific capacitance, energy density, and power density of the composite at different current densities.

Electrode	Current Density (A/g)	Specific Capacity (F/g)	Energy Density (Wh/kg)	Power Density (W/kg)	Electrolyte
JSPC&Ti_3_C_2_Tx-1	10	48.17	4.5	4100	1 M KOH
	5	57.55	8.98	2650	
	1	81.67	16.33	600	
	0.5	89.6	19.44	312.5	
JSPC&Ti_3_C_2_Tx (acetone)	10	58.17	8.74	5200	1 M KOH
	5	64.74	12.31	2925	
	1	86.99	18.28	615	
	0.5	96.83	21.35	315	
Pine sawdust [35]	0.5	175.6	24.39	254.06	1 M KOH
Bamboo stalk [30]	0.5	222	6.68	100.2	6 M KOH
Rice husk [36]	0.5	278	7.4	6195	6 M KOH
Cotton [37]	0.5	365	16.1–5.5	200–3700	6 M KOH
Chitosan [38]	0.5	317	48.6	468.8	6 M KOH

## Data Availability

The data presented in this study are available on request from the corresponding author.
